# Immune Response to an Adjuvanted Recombinant Zoster Vaccine in Japanese Patients with Rheumatoid Arthritis Receiving Upadacitinib (End Zoster-J Study): Study Protocol for an Exploratory Parallel Triple-Arm Prospective Trial

**DOI:** 10.3390/jcm13237321

**Published:** 2024-12-02

**Authors:** Ryu Watanabe, Hisako Fujii, Takumi Imai, Yuki Furusawa, Masao Katsushima, Kazuo Fukumoto, Yutaro Yamada, Kenji Mamoto, Tadashi Okano, Shinsuke Yamada, Motomu Hashimoto

**Affiliations:** 1 Department of Clinical Immunology, Graduate School of Medicine, Osaka Metropolitan University, Osaka 545-8585, Japan; 2 Department of Health and Medical Innovation, Graduate School of Medicine, Osaka Metropolitan University, Osaka 545-8585, Japan; 3 Department of Medical Statistics, Graduate School of Medicine, Osaka Metropolitan University, Osaka 545-8585, Japan; 4 AbbVie GK, Medical Affairs, Tokyo 108-0023, Japan; 5 Department of Orthopaedic Surgery, Graduate School of Medicine, Osaka Metropolitan University, Osaka 545-8585, Japan; 6 Center for Senile Degenerative Disorders, Medical School, Osaka Metropolitan University, Osaka 545-8585, Japan

**Keywords:** herpes zoster, methotrexate, rheumatoid arthritis, upadacitinib, vaccine

## Abstract

**Background**: Janus kinase (JAK) inhibitors have emerged as a new class of disease-modifying anti-rheumatic drugs in the treatment of rheumatoid arthritis (RA). However, herpes zoster is one of the common adverse events of JAK inhibitors, including upadacitinib, which is especially high in Japanese patients with RA compared to those from Western countries. Recombinant zoster vaccine (Shingrix^®^) is an adjuvanted subunit vaccine containing varicella-zoster virus (VZV) glycoprotein E (gE) that is effective in adults over 50 years of age. Despite this, no studies have examined its immunogenicity in Japanese patients receiving upadacitinib. Therefore, this study aims to examine the effectiveness of the recombinant zoster vaccine in Japanese patients with RA receiving upadacitinib. **Methods**: This is a single-center, exploratory, interventional, open-label, parallel triple-arm, prospective study. A total of 69 patients (23 in each group) aged 50 years or over and treated with a stable dose of methotrexate (MTX) monotherapy (6–12 mg/week), upadacitinib monotherapy (15 mg/day), or MTX (6–12 mg/week) + upadacitinib 15 mg/day (combination) for at least 1 month prior to study entry will be included. Moreover, all three groups will receive two intramuscular injections of the recombinant zoster vaccine at 8-week intervals. The primary endpoint is the proportion of positive anti-gE antibodies 4 weeks after the second injection. Secondary endpoints include RA disease activity, positive gE-specific CD4+ T-cells, and VZV-specific antibodies at indicated time points. Data on outcome measures will be collected at baseline and at 4, 8, 12, and 20 weeks. Endpoints will be summarized using descriptive statistics from baseline therapy, and results will be compared in an exploratory manner. **Discussion**: Despite the limited generalizability due to its design as a single-center, single-ethnic study, small sample size, and short observation period, this study provides evidence on the effectiveness and tolerability of recombinant zoster vaccine in Japanese patients with RA receiving upadacitinib.

## 1. Introduction

Rheumatoid arthritis (RA) is a chronic inflammatory disease causing progressive joint destruction and physical impairment if untreated [1]. Significant progress has been made in the treatment of RA over the decades. Conventional synthetic disease-modifying anti-rheumatic drugs (csDMARDs), such as methotrexate (MTX), and biological DMARDs, including tumor necrosis factor (TNF) inhibitors, interleukin (IL)-6 receptor antibodies, and abatacept, have become widely used for RA. Targeted synthetic DMARDs (tsDMARDs), also known as Janus kinase (JAK) inhibitors, have emerged as a new class of DMARDs. Currently, five JAK inhibitors (tofacitinib, baricitinib, upadacitinib, peficitinib, and filgotinib) are available in Japan, offering equivalent or superior effectiveness compared to existing bDMARDs [2]. This has made remission or low disease activity achievable goals in most patients. However, challenges remain for those who are refractory to any treatment, referred to as difficult-to-treat RA [3,4].

Herpes zoster is a common adverse event associated with JAK inhibitors, occurring in approximately 3–5% of patients with RA [5]. Particularly, Japanese patients with RA exhibit a higher incidence of herpes zoster compared to their European and American counterparts [5]. While recombinant zoster vaccine (Shingrix^®^), a subunit vaccine containing varicella-zoster virus (VZV) glycoprotein E (gE) with the AS01B adjuvant system, has been reported to be effective in adults over 50 years of age [6], JAK inhibitors (tofacitinib and baricitinib) partially attenuate its immunogenicity at 1.5 months post-administration [7]. This was consistent with a case report of a tofacitinib-treated patient who developed herpes zoster despite vaccination [8]. Furthermore, the vaccine is known to cause RA flares within 12 weeks after administration in some cases [8,9,10]. Nevertheless, studies investigating the immunogenicity of this vaccine in the presence of upadacitinib are limited, particularly among Japanese patients.

To address this gap, we aim to examine the immune response to the recombinant zoster vaccine Shingrix^®^ in Japanese patients with RA receiving upadacitinib. We assess immunogenicity at week 4 and RA flare ups to week 12 after the second Shingrix^®^ dose in patients receiving MTX (6–12 mg/week) monotherapy (Arm 1), upadacitinib (15 mg/day) monotherapy (Arm 2), and MTX (6–12 mg/week) + upadacitinib (15 mg/day) combination therapy (Arm 3). Immunogenicity and RA flares will be determined as previously described (immunogenicity [11] and RA flare [7,10]). In addition to humoral immune response (anti-gE-specific antibodies and anti-VZV-specific antibodies), cellular immune response (e.g., VZV-specific T-cell response) will be tested in this study, since the latter is a better surrogate marker for protection against herpes zoster [12,13,14]. Both humoral and cellular immunity have been reported to peak approximately 1 month after the second shot of the vaccine [15]; thus, the time point for the primary endpoint was set at 4 weeks after the second shot. In addition, the time points for secondary endpoints were set at 4 weeks after the first and second shots. Moreover, since recombinant zoster vaccine is associated with potential exacerbations or new onset of immune-mediated disease [16], not only disease activity of RA but also other adverse events will be carefully monitored.

The objective of this study is to investigate the immunogenicity and disease flares following recombinant zoster vaccination (Shingrix^®^) in Japanese patients with RA receiving MTX (6–12 mg/week) + upadacitinib (15 mg/day) combination therapy.

This is an exploratory, interventional, open-label, parallel triple-arm, prospective study. The three arms comprise the following regimens administered at patient registration:

(1) MTX monotherapy (6–12 mg/week).
(2) Upadacitinib monotherapy (15 mg/day).
(3) MTX (6–12 mg/week) + upadacitinib (15 mg/day) (primary evaluation target) combination therapy.

All patients in the three groups will receive two intramuscular injections of the recombinant zoster vaccine at 8-week intervals. It should be noted that MTX can be used up to 16 mg/week in Japan. However, the dose indicated in the study was based on the typical dose administered in most institutions (6–12 mg/week) [17,18]. The upadacitinib dose (15 mg/day) was set as the approved dose.

## 2. Methods

### 2.1. Study Setting

This is a single-center trial conducted at Osaka Metropolitan University Hospital, Osaka, Japan.

### 2.2. Trial Registration

This study was registered with the Japan Registry of Clinical Trials (jRCTs051220105) before enrolling the first participants (14 October 2022).

### 2.3. Eligibility Criteria

The eligibility criteria include the following: (1) fulfilled the 1987 or 2010 classification criteria for RA [19,20]; (2) aged over 50 years; (3) provided written informed consent; (4) received MTX monotherapy (6–12 mg/week), upadacitinib monotherapy (15 mg/day), or MTX (6–12 mg/week) + upadacitinib (15 mg/day) combination therapy for at least 1 month prior to the study entry; (5) and able to adhere to follow-ups based on the research schedule. Patients with prior exposure to JAK inhibitors other than upadacitinib may be enrolled (up to 20% of the total study population) if they responded to previous JAK inhibitors but had to discontinue treatment due to adverse events (1-week wash out period is required prior to the first dose of the study drug).

The exclusion criteria include the following: (1) required hemodialysis; (2) pregnant or lactating; (3) had severe liver dysfunction (Child–Pugh C); (4) had serious infection or active tuberculosis; (5) presented with neutropenia (<1000/mm^3^), lymphopenia (<500/mm^3^), or anemia (haemoglobin <8 g/dL); (6) previously received recombinant zoster vaccine (Shingrix^®^); (7) previously received a live herpes zoster vaccine within 8 weeks before enrollment; (8) developed herpes zoster or had related symptoms within 6 months before enrollment; (9) received any vaccine within 4 weeks before enrollment or planned to receive other vaccines (except Shingrix^®^) between enrollment and 4 weeks after the second shot of Shingrix^®^; (10) congenital or acquired immunodeficiency; (11) received prednisolone (equivalent) >10 mg; (12) discontinued prior JAK therapy due to insufficient efficacy; and (13) otherwise deemed unsuitable for participation in this study.

### 2.4. Who Will Take Informed Consent?

The principal investigator or co-investigator will obtain written informed consent.

### 2.5. Additional Consent Provisions for Collection and Use of Participant Data and Biological Specimens

An additional 6 mL of peripheral blood will be drawn from the enrolled patients. Serum, peripheral blood mononuclear cells (PBMCs), and RNA extracted from PBMCs will be stored for ancillary research.

## 3. Interventions

### 3.1. Explanation for the Choice of Comparators

The study will employ two comparator groups to assess the effect of the recombinant zoster vaccine (Shingrix^®^): MTX monotherapy (6–12 mg/week) and upadacitinib monotherapy (15 mg/day).

### 3.2. Intervention Description

**Figure 1:**
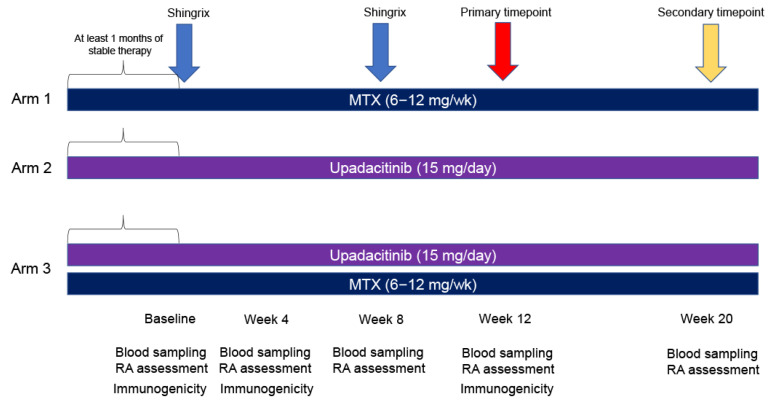
Overview of the study.

All patients across the three arms will receive two intramuscular injections of the recombinant zoster vaccine (Shingrix^®^) at 8-week intervals (Figure 1).

### 3.3. Criteria for Discontinuing or Modifying Allocated Interventions

#### 3.3.1. Criteria for Discontinuing Allocated Interventions

Allocated interventions are discontinued for the following situations:

(1) When the patient withdraws the informed consent.
(2) When a principal investigator or co-investigator judges that it is difficult to continue the research due to worsening of the primary disease, complications, or the occurrence of serious adverse events.
(3) When there is a need to administer contraindicated drugs.
(4) When serious deviations from the research protocol occur, such as violations of the Clinical Research Act and its implementation regulations, inclusion, or exclusion criteria. Examples of serious deviations are listed below, but are not limited to these events:
  - Medication non-compliance: interruption of oral medication for 14 consecutive days.
  - Patients who did not receive at least one dose of Shingrix^®^ as scheduled.
  - Patients who received other vaccines between obtaining informed consent and 4 weeks after the second Shingrix^®^ dose.
(5) When pregnancy of the study subject is discovered.
(6) When the study is discontinued.
(7) When the drugs being administered (MTX and Upadacitinib) are discontinued or are found to have been discontinued prior to intervention.
(8) When the principal investigator or co-investigator determines that it is difficult to continue the study.

#### 3.3.2. Criteria for Modifying Allocated Interventions

The Shingrix^®^ dosing interval can be extended up to 10 weeks.

### 3.4. Strategies to Improve Adherence to Interventions

At each visit, the principal investigator and co-investigators monitor drug adherence, adverse events, laboratory tests, and the date of the next scheduled visit.

### 3.5. Relevant Concomitant Care Permitted or Prohibited During the Trial

Concomitant use of other anti-rheumatic drugs, analgesics, and necessary medications (except other bDMARDs and JAK inhibitors) is permitted. However, dose changes of anti-rheumatic drugs, including MTX, upadacitinib, and glucocorticoids, are prohibited from the time of consent acquisition until 4 weeks after the second Shingrix^®^ dose.

### 3.6. Provisions for Post-Trial Care

Compensation for those who suffer harm from study participation will be provided up to 12 months post-disease/injury, based on consultation with the principal investigator, medical insurance company, and other relevant departments. The amount of compensation will be determined based on the severity of the adverse events.

Further ancillary research will be conducted after the research plan has been approved by the Ethics Committee, and patient consent will be obtained prior to its implementation.

## 4. Outcomes

### 4.1. Primary Endpoint

Proportion of participants with positive anti-gE antibodies at 4 weeks after the second Shingrix^®^ dose.

### 4.2. Secondary Endpoints

(1) Presence or absence of RA flares from enrollment to 12 weeks after the second Shingrix^®^ dose.
(2) Proportion of participants with positive anti-gE antibodies at 4 weeks after the first Shingrix^®^ dose.
(3) Antibody titre of anti-gE antibodies at 4 weeks after the first and second Shingrix^®^ doses.
(4) Proportion of gE-specific CD4+ T-cells at 4 weeks after the first and second Shingrix^®^ doses.
(5) Antibody titre of VZV-specific antibodies at 4 weeks after the first and second Shingrix^®^ doses.
(6) Changes in RA disease activity.

### 4.3. Evaluation of Primary and Secondary Endpoints

#### 4.3.1. Positive Anti-gE Antibodies

This is defined as having an antibody titre 4 weeks after the first or second Shingrix^®^ doses that is increased 4 or more times from baseline (before Shingrix^®^ injection at week 0) or the cutoff value calculated, as in previous reports [11]. The time points for this assessment were determined based on a previous study [11]. Anti-gE antibodies will be evaluated using an enzyme-linked immunosorbent assay (ELISA) by AbbVie Deutschland GmbH and Co.

#### 4.3.2. RA Flare

This is defined based on the following: (1) judgment by the attending physician, (2) administration of new or increased dose of glucocorticoids, (3) increased or addition of anti-rheumatic drugs, and (4) increased disease activity based on a Disease Activity Score (DAS)28-C-reactive protein (CRP) ≥0.6. The observation period was set until 12 weeks after the second Shingrix^®^ dose based on previous studies [7,10]. However, an extension of 2 weeks (20 -2 to +4 weeks from the enrollment of the study) is allowed.

#### 4.3.3. Proportion of gE-Specific CD4+ T-Cells

This is defined with gE-specific CD4+ T-cells expressing ≥ 2 of the 4 activation markers: IFN-γ, IL-2, TNF-α, and CD40 ligand. Assessment will be performed using flow cytometry at Labcorp Central Laboratory Services Limited Partnership, Labcorp Translational Biomarker Solutions. The proportion of gE-specific CD4+ T-cells was calculated as follows: gE-specific CD4+ T-cells/total CD4+ T-cells.

#### 4.3.4. Antibody Titre of VZV-Specific Antibodies

Evaluation of VZV-specific antibodies will be performed using ELISA at the Osaka Metropolitan University.

#### 4.3.5. Changes in RA Disease Activity

RA disease activity will be monitored using the DAS28-CRP, DAS28-erythrocyte sedimentation rate (DAS28-ESR), simplified disease activity index (SDAI), and clinical activity index (CDAI).

If flare is observed, treatment will be at the discretion of each attending physician and will not be limited by this study (Table 1).

### 4.4. Sample Size

Given the design as an exploratory study, the sample size was calculated based on the number of patients at our hospital. In our institution, approximately 1000 patients with RA are being treated, including 300 who are currently receiving MTX monotherapy and 30 who are receiving upadacitinib.

**Figure 2:**
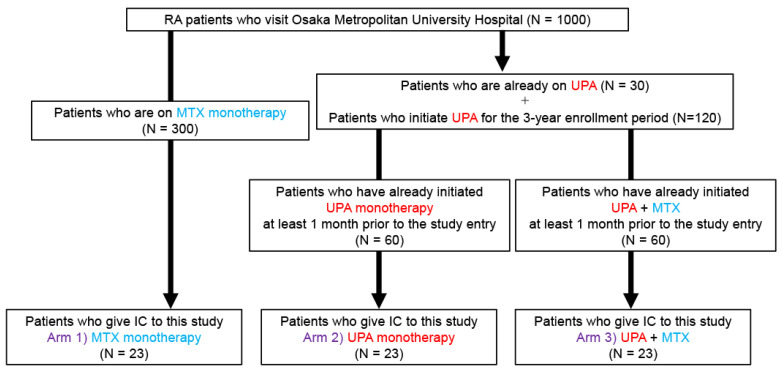
Sample size explanation. IC: informed consent, MTX: methotrexate, RA: rheumatoid arthritis, UPA: upadacitinib.

An average of 80 patients receive JAK inhibitors per year, including 40 patients who have initiated upadacitinib therapy (with or without MTX). Therefore, 120 upadacitinib initiations were projected for the 3-year enrollment period. Accordingly, informed consent can be obtained from 40 patients for each group (20 in the MTX combination group, 20 in the upadacitinib monotherapy group). Accounting for a 10% dropout rate, the target enrollment was set at 23 patients for each group (Figure 2).

### 4.5. Recruitment

Eligible patients will be recruited from our hospital. In cases of participant shortage, patients can be recruited from nearby hospitals and clinics.

## 5. Assignment of Interventions: Allocation

### 5.1. Sequence Generation

Not applicable to this study.

### 5.2. Concealment Mechanism

Not applicable to this study.

### 5.3. Implementation

All patients will be assigned to receive Shingrix^®^.

### 5.4. Who Will Be Blinded

The treatment arm of each participant is not disclosed to the outcome assessors for blinded assessment of anti-gE antibodies or gE-specific CD4+ T-cells.

### 5.5. Procedure for Unblinding if Needed

Not applicable to this study.

## 6. Data Collection and Management

### 6.1. Plans for Assessment and Collection of Outcomes

Study data will be collected and managed using REDCap (Research Electronic Data Capture) electronic data capture (EDC) tools hosted at Osaka Metropolitan University. REDCap is a secure, web-based software platform designed to support data capture for research studies, providing (1) an intuitive interface for validated data capture; (2) audit trails for tracking data manipulation and export procedures; (3) automated export procedures for seamless data downloads to common statistical packages; and (4) procedures for data integration and interoperability with external sources.

Humoral and cell-mediated immunogenicity data will be electronically provided by AbbVie Deutschland GmbH and Co KG and the Labcorp Central Laboratory Services Limited Partnership, respectively.

### 6.2. Plans to Promote Participant Retention and Complete Follow-Up

To promote participant retention and complete follow-up, the principal investigator and co-investigators will hold regular meetings once a month to discuss participants who discontinue or deviate from the protocol.

### 6.3. Data Management

All collected data will be stored by the principal investigator or co-investigator using the password-protected EDC system. Data will be double-checked by dedicated monitoring personnel.

### 6.4. Confidentiality

Personal identifiers, including names, initials, and patient IDs, will be removed. Anonymized correspondence tables with new codes will be generated to ensure participant anonymity. These tables will be stored on a password-locked standalone computer, disconnected from the internet and the hospital’s electronic medical record network. Additionally, the principal investigator will store documents related to the study in a lockable storage cabinet for at least 5 years after study completion.

Upon disposal of relevant materials after the storage period, data will be erased from the computer, and paper documents will be disposed using a shredder.

When sending blood samples to research institutions for the evaluation of immunogenicity, only the new code, age, and sex registered in the EDC system will be provided. Details of the treatment will be blinded to the institutions to avoid bias.

### 6.5. Plans for Collection, Laboratory Evaluation, and Storage of Biological Specimens for Genetic or Molecular Analysis in This Trial/Future Use

An additional 6 mL of peripheral blood will be collected, and serum, PBMCs, and RNA extracted from PBMCs will be stored. Currently, we plan to analyze the gene expression related to the pathophysiology of RA and inflammation as an ancillary study into this research. Ancillary research will be conducted once approval from the Ethics Committee and patient consent are obtained.

## 7. Statistical Methods

### 7.1. Statistical Methods for Primary and Secondary Outcomes

#### 7.1.1. Full Analysis Set (FAS)

FAS includes patients who received at least one Shingrix^®^ dose.

#### 7.1.2. Per Protocol Set (PPS)

From the FAS, PPS excludes patients who have seriously violated the protocol, as indicated below:

- Violation of the inclusion criteria.
- Exclusion criteria violations.
- Medication compliance violation (interruption of oral medication for 14 consecutive days).
- Failure to receive Shingrix^®^ injections.
- Injection of other vaccines between the time of consent and 4 weeks after the second Shingrix^®^ dose.
- Dose changes of anti-rheumatic drugs (e.g., MTX, glucocorticoids, upadacitinib) between the time of consent and 4 weeks after the second Shingrix^®^ dose.

#### 7.1.3. Safety Analysis Set (SAS)

SAS includes patients who received at least one Shingrix^®^ dose.

### 7.2. Primary Endpoint

The number and proportion of patients positive for anti-gE antibodies at 4 weeks after the second Shingrix^®^ dose will be summarized for each group.

### 7.3. Secondary Endpoints

(1) Presence or absence of RA flares from enrollment to 12 weeks after the second Shingrix^®^ dose.

The numbers and proportions of patients with RA flares are summarized for each group.

(2) Proportion of positive anti-gE antibodies at 4 weeks after the first Shingrix^®^ dose.

The number and proportion of patients positive for anti-gE antibodies at 4 weeks after the first Shingrix^®^ dose will be summarized for each group. Evaluations will also be conducted after adjusting for confounders.

(3) Antibody titre of anti-gE antibodies at 4 weeks after the first and second Shingrix^®^ doses.

Means, standard deviations, medians, interquartile ranges, minimum values, maximum values, 95% confidence intervals, and numbers of missing values are described for each group.

(4) Proportion of gE-specific CD4+ T-cells at 4 weeks after the first and second Shingrix^®^ doses.

Means, standard deviations, medians, interquartile ranges, minimum values, maximum values, 95% confidence intervals, and numbers of missing values are described for each group.

(5) Antibody titer of VZV-specific antibodies at 4 weeks after first and second Shingrix^®^ doses.

Means, standard deviations, medians, interquartile ranges, minimum values, maximum values, 95% confidence intervals, and numbers of missing values are described for each group.

(6) Changes in RA disease activity.

Means, standard deviations, medians, interquartile ranges, minimum values, maximum values, 95% confidence intervals, and numbers of missing values are described for each group.

### 7.4. Interim Analyses

Interim analyses will not be conducted in this study.

### 7.5. Methods for Additional Analyses (e.g., Subgroup Analyses)

Multivariate logistic regression with adjustments for confounding factors (age, sex, and disease severity) will be performed as a supplementary analysis. The study subject background will be reviewed, and the addition or exclusion of confounding factors will be allowed under blinding of anti-gE antibody data.

### 7.6. Methods in Analysis to Handle Protocol Non-Adherence and Any Statistical Methods to Handle Missing Data

#### 7.6.1. Handling of Patients Enrolled in the Study

In case of a query, the handling of patients will be decided after a discussion between the principal investigator, statistical analyst, and other relevant departments. Decisions regarding case handling are also recorded.

#### 7.6.2. Handling of Outliers or Abnormal Values

If it is an obvious error, values will be reconsidered or remeasured; otherwise, no changes will be made to these values.

#### 7.6.3. Handling of Missing Values

Omissions in the EDC system are monitored and compensated, if necessary. No multiple imputations will be performed. Missing values outside the EDC system can be compensated at the next visit, if possible.

### 7.7. Plans to Give Access to the Full Protocol, Participant Level-Data, and Statistical Code

Access to the protocol and other materials related to the study will be provided to the research subjects upon request.

## 8. Oversight and Monitoring

### 8.1. Composition of the Coordinating Centre and Trial Steering Committee

The Center for clinical research and innovation (CCRI) at Osaka Metropolitan University Hospital will assist in protocol development, EDC system setup, statistical analysis, monitoring, data management, and manuscript editing. The principal investigator will maintain contact with the study participants at least once a month.

### 8.2. Composition of the Data Monitoring Committee, Its Role and Reporting Structure

For quality control, the principal investigator will prepare a written procedure for monitoring, which will be approved by the accredited clinical research review committee. Moreover, the principal investigator will designate a person in charge of monitoring, independent of the sponsor and competing interests. Throughout the study period, the person in charge of monitoring will confirm if the study is being conducted in accordance to the monitoring protocol, as well as the latest guidelines and regulatory requirements (e.g., Clinical Research Act, enforcement regulations). Furthermore, this person will report the results to the principal investigator in accordance with the monitoring protocol. In addition, any personal information of the research participants obtained from monitoring should not be divulged.

### 8.3. Adverse Event Reporting and Harms

The time of onset/disappearance, treatment, outcome, severity, relationship with study drugs, and predictability of adverse events will be documented in the medical records and EDC system. All adverse events occurring from study drug initiation to the end of the observation period will be recorded.

#### 8.3.1. Definition of Adverse Events

An adverse event is defined as any unfavourable symptom, sign, disease, or abnormal laboratory test value that occurs in a patient after administration of the study drug, regardless of the causal relationship.

#### 8.3.2. Evaluation for Severity

The severity of adverse events is evaluated according to the following criteria:

(1) Mild: when administration can be continued without treatment.
(2) Moderate: when administration can be continued with some kind of treatment.
(3) Severe: when discontinuation is required for the patient.

#### 8.3.3. Evaluation for Seriousness

Seriousness is determined according to the following criteria:

(1) Serious
  1. Death.
  2. A condition that may lead to death.
  3. A condition that requires hospitalization at a medical institution or an extension of hospitalization for treatment.
  4. Disability.
  5. A condition that may lead to disability.
  6. A condition that is serious according to criteria 1–5.
  7. Congenital diseases or abnormalities in later generations.

Note: Hospitalizations scheduled before study participation, hospitalizations for examination purposes, and extension of the hospitalization period are not considered serious adverse events.

(2) Non-Serious

Anything other than “serious” aforementioned events.

#### 8.3.4. Illness Related to the Study

(1) Definition of illness

Disease, disability, death, infectious disease, or abnormal laboratory test values and symptoms suspected to be caused by the implementation of the study are defined as illnesses. Moreover, a causal relationship between the study drug used and the study procedure will be determined by the principal investigator and co-investigator.

(2) Predictability

Events that cannot be predicted from the package insert (container/encapsulation) of the study drug and the precautions written in the interview form are categorized as “unknown,” whereas those that are listed and can be predicted are categorized as “known”.

(3) Causality

In this study, causality is defined as a causal relationship that cannot be denied, based on the judgement of the principal investigator and co-investigator. When deciding, they will consider not only the temporal relationship with the initiation of interventional but also the course of underlying diseases, complications, concomitant medications, study procedures, accidents, and other environmental factors. Causality is judged and recorded throughout the study period.

#### 8.3.5. Subjects and Reporting Period for Reporting Illness

If the investigator becomes aware of a disease or other matters that requires reporting within the time limit listed in the table below, the principal investigator reports it to the administrator of the medical institution and an accredited clinical research review board within the specified period. Additionally, this information will be provided to manufacturers and distributors of pharmaceuticals (Table 2).

#### 8.3.6. Procedures for Reporting Illnesses: Procedures in the Event of Illness

(1) Co-investigators report the event to the principal investigator if they become aware of the illness. The principal investigator and co-investigators will then take appropriate measures for the study subject, take the best care, and record it in the medical records.
(2) If the illness is subject to reporting within the deadlines listed in Table 1, the investigators must submit a report to the accredited clinical research review board within the specified reporting deadline.

#### 8.3.7. Procedures for Reporting Illnesses: Procedures of Regular Reporting

(1) The principal investigator will use the jRCT system to report the number of illnesses to the Minister of Health, Labor, and Welfare.
(2) The principal investigator will report the number of illnesses to the administrator of the medical institution and to the accredited clinical research review board within the period specified in Table 1.
(3) With the approval of the accredited clinical research review committee, the principal investigator will use the jRCT system to report to the Regional Bureau of Health and Welfare within the period specified in Table 1.

#### 8.3.8. Observation of Research Subjects After Occurrence of Adverse Events

Follow-up surveys of the study subjects after the occurrence of adverse events will be conducted until resolution of adverse events or until the principal investigator or co-investigator determines that follow-up surveys are unnecessary.

### 8.4. Frequency and Plans for Auditing Trial Conduct

Audits will be conducted as necessary in accordance with the regulations of the Clinical Research Act.

### 8.5. Plans for Communicating Important Protocol Amendments to Relevant Parties (E.G., Trial Participants, Ethical Committees)

(1) When amending the study protocol and informed consent form, the principal investigator must obtain a review from an accredited clinical research review committee prior to the amendment and report the details to the administrator of the implementing medical institution. In the case of changes related to the implementation system or other significant changes, approval from the administrator of the implementing medical institution should be obtained, as necessary. The principal investigator or co-investigator must not conduct the study using a modified research protocol or informed consent form before obtaining approval from the committee or the administrator of the implementing medical institution.
(2) When changes in the study protocol involve changes in the implementation plan, the principal investigator must submit a notification of changes to the Minister of Health, Labor, and Welfare after obtaining approval from the accredited clinical research review committee. The principal investigator or co-investigators must not conduct the study using the modified study protocol and informed consent form before submitting the notification of modification to the Minister of Health, Labor, and Welfare, and before such modification is published in the jRCT.

### 8.6. Dissemination Plans

Communicating the trial results to participants, healthcare professionals, and the public will be done through domestic and international conference presentations and publications.

## 9. Discussion

This study aims to investigate the immune response to the recombinant zoster vaccine Shingrix^®^ in Japanese patients with RA receiving MTX + upadacitinib combination therapy, MTX monotherapy, and upadacitinib monotherapy. To the best of our knowledge, this is the only prospective study conducted in Japan examining the efficacy and safety of this vaccine in this cohort. This study could provide pivotal evidence in Japan, where the incidence of herpes zoster is relatively higher. Compared to a previous US study of this vaccine in the same population [21], the present study’s parallel triple-arm design offers an advantage in presenting validated results.

Another strength of this study is that it examines both humoral (e.g., anti-gE, VZV-specific antibodies) and cellular (e.g., gE-specific CD4+ T-cells) immunity, since cellular immunity is considered a more reliable predictor of herpes zoster protection [12,13,14]. Furthermore, ancillary RNA sequencing will be performed using PBMCs, allowing the analysis of gene expression before and after vaccination. Such a comprehensive approach will allow for a multifaceted examination of vaccine immunogenicity. If these studies prove the efficacy and safety of the vaccine, it will provide beneficial insights into herpes zoster prevention strategies for patients receiving JAK inhibitors, not only in Japan but also in other countries.

Despite the insights offered in our study, several limitations should be acknowledged. First, this is a single-center prospective study that will only enroll Japanese patients with RA. Therefore, the results cannot be generalized globally. Second, due to the study’s exploratory design, the sample size is relatively small (target of 23 patients per group) in consideration to feasibility. Third, the primary endpoint in this study was set at 4 weeks after the second Shingrix^®^ dose, which was based on a previous study [11]. However, other studies have evaluated the immunogenicity of the vaccine up to 10 years [22,23]. Thus, this study only evaluates the short-term immunogenicity of the vaccine. Lastly, we will not examine the incidence of herpes zoster due to the short observation period. To verify the effectiveness of the vaccine, further studies must include a larger number of patients with longer-term observations [6,24].

Nevertheless, this study will provide critical evidence on Shingrix^®^ effectiveness and tolerability in Japanese patients with RA receiving upadacitinib, which is particularly relevant given the nation’s high incidence of herpes zoster. Until recently, the recombinant zoster vaccine has been limited to individuals aged 50 years and over; however, individuals aged 18 years and over who are considered to be at high risk of herpes zoster have been eligible for vaccination in Japan since June 2023. Furthermore, upadacitinib has been indicated for other diseases that develop at a relatively young age, including ankylosing spondylitis, atopic dermatitis, ulcerative colitis, non-radiographic axial spondyloarthritis, and Crohn’s disease. As such, the study provides useful evidence for these cohorts.

## 10. Trial Status

The date the protocol was approved: 29 September 2022 (version 1.0).

The date recruitment began: 14 October 2022.

The date first patient registered: 26 December 2022.

The date the protocol was revised: 26 Oct 2023 (version 2.3).

The date the revised protocol was approved: 28 November 2023.

Trial status: Recruiting.

The approximate date when recruitment will be completed: 31 October 2024.

The observation period: From 12 October 2022 to 31 March 2025.

The estimated date for study completion: 31 March 2026.

## Figures and Tables

**Table 1:** Participant timeline.

	Before Enrollment	At Enrollment	Week 4	Week 8	Week 12	Week 20
Visit	0	1	2	3	4	5
Allowance			+1 week	+2 week	+3 week * 1	−2~+4 week * 2
Informed consent		X				
Eligibility screen		X				
Shingrix^®^ injection		X		X		
Disease activity of RA	X	X	X	X	X	X
Assessment of adverse events		X	X	X	X	X
Blood tests * 3 Urine tests * 4 Serum storage	X	X	X	X	X	X
Immunogenicity * 5		X	X		X	
Additional blood collection		X	X		X	
Confirmation of concomitant drug	X	X	X	X	X	X
RA flare						X

* 1: 4 weeks + 1 week after the second Shingrix^®^ dose; * 2: 12 weeks ± 2 weeks after the second Shingrix^®^ dose; * 3: Blood tests include white blood cell count, red blood cell count, haemoglobin, platelet count, albumin, aspartate aminotransferase (AST), alanine aminotransferase (ALT), creatinine, blood urea nitrogen (BUN), CRP, and rheumatoid factor (RF); * 4 Urine test results include urine protein and occult blood; * 5 Immunogenicity evaluation includes anti-gE antibody, gE-specific CD4+ T-cells, and VZV-specific antibodies.

**Table 2:** Type of report.

			Regional Bureau of Health and Welfare	Accredited Clinical Research Review Board	
Type of Report			Regular Report	On-Time Report	Regular Report
Pharmaceuticals	Known	Death	〇	15 days	〇
		Serious condition other than death	〇	15 days	〇
		Non-serious	〇	―	〇
	Unknown	Death	〇	15 days	〇
		Serious condition other than death	〇	30 days	〇
		Non-serious	〇	―	〇
Infection	Known	Death/Serious	〇	15 days	〇
		Non-serious	〇	15 days	〇
	Unknown	Death/Serious	〇	15 days	〇
		Non-serious	〇	―	〇

〇 means required, and ― means not required.

## Data Availability

The dataset used and analyzed in the current study are available from the corresponding author upon reasonable request.

## Abbreviations

AST	aspartate aminotransferase
ALT	alanine aminotransferase
BUN	blood urea nitrogen
CDAI	clinical disease activity index
CRP	C-reactive protein
DAS	disease activity score
ESR	erythrocyte sedimentation rate
gE	glycoprotein E
JAK	Janus kinase
jRCT	Japan Registry of Clinical Trials
RA	rheumatoid arthritis
SDAI	simplified disease activity index
VZV	varicella-zoster virus
